# Japanese Nursery Notes

**Published:** 1893-09

**Authors:** 


### Japanese Nursery Notes.

It has often been said that the Japanese are the most interesting, the strangest, even the quaintest, people we know. In no regard is this truer than in the care they take of their babies. Such a strong foundation is more necessary than elsewhere to a nation where man is born to remain a baby his whole life. We, destined to exercise stronger and more serious minds, would be, at the very beginning of our existence, deteriorated by such ingenious untiring care.

I have spoken, in another article, of the long-continued lactation of Japanese women as benefiting both mother and child; also of the care taken of pregnant women, in which a solicitude displays itself at the same time clever and loving. These tender and intelligent attentions paid to the born baby is the second part of that uinque Japanese system of rearing healthy and happy men, which makes European and American ladies forget that so many other Japanese conceits are a severe shock to their feelings.

During the dentition period the children have an extra diet, consisting of fish and small crustacea. Japanese not being a carnivorous people, this is natural enough. If they ate meat they would give their children beef very likely. But it is certainly to the advantage of the bony structure of the child to be, on first entering the adult course of eating, fed in the Japanese manner.

There is a singular difference between the carriage of Japanese children and the way in which our children walk and move about. The Japanese urchin, whose feet never knew the unkind pressure of tight shoes, and, in fact, no pressure at all, walks more erect, is more sure-footed. In fair weather he wears flat shoe sandals; in these sandals the big toe is widely separated from the others, which gives the child a surer foundation. In wet weather he must maintain his equilibrium on his stilt-like wooden clogs, which keep his feet dry, at the same time compelling him to acquire an extraordinary power over his own motions.

There is in Japan no kissing, not even in the nursery. All the dangers which have been so eloquently described in newspapers some time ago, arising from the touch of lips, in human love directly, and at the communion table indirectly, are avoided by the national aversion for labial contact.—*University Medical Magazine.*

